# Mutational analysis of ribosomal proteins in a cohort of pediatric patients with T-cell acute lymphoblastic leukemia reveals Q123R, a novel mutation in RPL10

**DOI:** 10.3389/fgene.2022.1058468

**Published:** 2022-11-22

**Authors:** Lorenza Bacci, Valentina Indio, Guglielmo Rambaldelli, Cristina Bugarin, Franco Magliocchetti, Alberto Del Rio, Daniela Pollutri, Fraia Melchionda, Andrea Pession, Marina Lanciotti, Carlo Dufour, Giuseppe Gaipa, Lorenzo Montanaro, Marianna Penzo

**Affiliations:** ^1^ Department of Experimental, Diagnostic and Specialty Medicine (DIMES), University of Bologna, Bologna, Italy; ^2^ Center for Applied Biomedical Research (CRBA), University of Bologna, Bologna, Italy; ^3^ Department of Veterinary Medical Sciences, University of Bologna, Bologna, Italy; ^4^ Tettamanti Research Center, M. Tettamanti Foundation, Pediatric Clinic, University of Milano Bicocca, Monza, Italy; ^5^ Institute of Organic Synthesis and Photoreactivity (ISOF), National Research Council (CNR), Bologna, Italy; ^6^ Innovamol Consulting Srl, Modena, Italy; ^7^ Pediatric Oncology and Hematology Unit “Lalla Seràgnoli”, IRCCS AOU S.Orsola di Bologna, Bologna, Italy; ^8^ Department of Medical and Surgical Sciences (DIMEC), University of Bologna, Bologna, Italy; ^9^ Hematology Unit—IRCCS Istituto Giannina Gaslini, Genoa, Italy; ^10^ Departmental Program of Laboratory Medicine, IRCCS Azienda Ospedaliero-Universitaria di Bologna, Bologna, Italy

**Keywords:** ribosome, RPL10 mutation, translation, next generation sequencing—NGS, leukemia, Q123R, uL16 (RPL10)

## Abstract

T-cell acute lymphoblastic leukemia (T-ALL) is a subtype of ALL involving the malignant expansion of T-cell progenitors. It is driven by a number of different possible genetic lesions, including mutations in genes encoding for ribosomal proteins (RPs). These are structural constituents of ribosomes, ubiquitous effectors of protein synthesis. Albeit the R98S mutation in RPL10, recurring with a higher frequency among RP mutations, has been extensively studied, less is known about the contribution of mutations occurring in other RPs. Alterations affecting translational machinery may not be well tolerated by cells, and there may be a selective pressure that determines the emergence of mutations with a compensatory effect. To explore this hypothesis, we sequenced the exomes of a cohort of 37 pediatric patients affected by T-ALL, and analyzed them to explore the co-occurrence of mutations in genes involved in ribosome biogenesis (including RPs) and translational control, and in known T-ALL driver genes. We found that some of the mutations in these sub-classes of genes tend to cluster together in different patients, indicating that their co-occurrence may confer some kind of advantage to leukemia cells. In addition, our sequencing highlighted the presence of a novel mutation in RPL10, namely the Q123R, which we found associated with a defect in protein synthesis. Our findings indicate that genetic alterations involving ribosome biogenesis and translational control should be carefully considered in the context of precision medicine in T-ALL.

## Introduction

Acute lymphoblastic leukemia (ALL) is the most frequent pathology among childhood cancers, accounting for about 25% of all childhood cancer types (Nigro, 2013). Even though the mechanisms of ALL pathogenesis are not clearly defined yet, it is believed that genetic and environmental factors may add up to create a leukemia-prone setting (Pui et al., 2015). About 15% of ALLs are represented by T-cell ALL (T-ALL), where malignant clones express T-lineage markers (O’Brien et al., 2015). T-ALL is considered as a model for the multi-step nature of cancer onset and development, driven by different genetic lesions, leading to deregulation of cell growth, proliferation, survival, and differentiation during thymocyte development (van Vlierberghe and Ferrando, 2012). The most frequent lesions can be categorized as chromosomal translocations, duplications and deletions of DNA, deregulated gene expression, and point mutations (Hunger and Mullighan, 2015). All these different genetic abnormalities ultimately lead to one of the following: i) ectopic expression of oncogenic transcription factors; ii) constitutive activation of NOTCH1 signaling; iii) deregulation of cell cycle regulators; and iv) hyper-activation of cytokine signaling (de Bie et al., 2018). In addition, different mutational studies performed in the last decade on pediatric T-ALL revealed a relatively high frequency (close to 10%) of somatic mutations in genes encoding for ribosomal proteins (RPs), namely ribosomal protein L5 (RPL5), RPL10 (de Keersmaecker et al., 2013), RPL11 (Tzoneva et al., 2013) and RPL22 (Rao et al., 2012), and these genes are recognized as potential T-ALL driver genes (de Keersmaecker et al., 2013; Liu et al., 2017). These proteins are structural components of ribosomes, ubiquitous nanomachines deputed to protein synthesis. Ribosomes are composed of a large (L) and a small (S) subunit, collectively made up of 81 RPs (named RPLs for the large and RPSs for the small subunit) and 4 ribosomal RNAs (rRNAs) with a precise stoichiometry. A mounting body of evidence indicates that inherited or acquired alterations of ribosome components (either proteins or RNAs) are directly linked to cancer (Stumpf and Ruggero, 2011; Sulima and de Keersmaecker, 2017). In infant T-ALL, mutations occurring in RPs are found most frequently in RPL10 gene, being arginine 98 (R98) a mutational hotspot (de Keersmaecker et al., 2013). RPL10 plays a key role both in the final steps of ribosome assembly (being located at the subunits interface), and in the protein synthetic activity, being a constituent of the peptidyl-transferase center, the catalytic core of ribosomes (Pollutri and Penzo, 2020). It is therefore not surprising that the most frequent RPL10 mutation in T-ALL, R98S, alters the translation of specific targets, ultimately impacting leukemic cells metabolism (Kampen et al., 2019b) and resistance to pro-apoptotic stimuli (Kampen et al., 2019a), thus conferring an advantage to mutant cells.

Although the contribution to pediatric T-ALL of R98S RPL10 mutation has been extensively studied, the same cannot be said for other mutations occurring in RPL10 itself or in other RPs, possibly because they do not show up with a high frequency. Nonetheless, it would be helpful to acquire a broader picture of how altered ribosomes mechanistically contribute to the progression of T-ALL. To this end, we decided to study the occurrence of RP mutations in a cohort of patients with a diagnosis of T-ALL, and to correlate it to the presence of other mutations occurring in factors involved in ribosome biogenesis or protein synthesis control.

## Methods

### Patients and leukemia samples

This study included 37 T-ALL patients recruited at IRCCS Azienda Ospedaliero-Universitaria di Bologna, IRCCS Istituto Giannina Gaslini in Genoa and Ospedale Fondazione MBBM/Clinica Pediatrica Università Milano-Bicocca in Monza. The study was approved by the local Institutional Ethical Committees (Protocol numbers: Bologna EM369-2021 496/2018/Sper/AOUBo, Genoa 0032707/21, Monza FMBBM/USC/2022/19). The patients were recruited prospectively and retrospectively upon collection of the informed consent. The patients’ characteristics are reported in Supplementary Tables S1, S2. Tumor samples were obtained from peripheral blood (PB) or bone marrow (BM) aspirate. Matched non-tumor samples (N = 5) were obtained from PB at remission or from buccal swabs.

### DNA extraction, libraries preparation, sequencing and data analysis

Genomic DNA was extracted from blood samples and buccal swabs using the QIAamp DNA Mini Kit (Qiagen) according to the manufacturer’s instructions. Whole exome sequencing (WES) was performed on the DNA purified from tumor samples and selected remission samples. DNA libraries were constructed using the DNA Illumina Prep with enrichment (Illumina) according to the manufacturer’s instructions. Paired-end libraries were sequenced at 2 × 79 read length on a NextSeq 500 using the Mid Output kit (Illumina).

Data were analyzed by an open source bioinformatic pipeline implemented on Centos7 Server. Genetic variants with a frequency higher than 1% in healthy populations were filtered out based on GnomAD database (Karczewski et al., 2020). Single nucleotide variants, insertions and deletions were called with the Mutect2 function of GATK and then annotated with Annovar. The non-silent variants were selected and considered as “high confidence” according with these criteria: 1) depth of coverage > 15; 2) ratio > 0.2 (ratio = alternated allele depth of coverage/total depth of coverage); 3) Mutect2 quality filter = PASS. Rare and novel variants were also highlighted based on the frequency reported on human variability databases.

### Sanger validation

Mutations in RP encoding genes were confirmed by Sanger sequencing on diagnosis samples. When confirmed, Sanger sequencing was subsequently performed on matched remission samples/buccal swabs to address their germline/somatic origin. Only for those samples in which a somatic status was called, NGS analysis was subsequently performed on remission samples/buccal swabs. Primer sequences are reported in Supplementary Table S3.

### 3D model

The RPL10 structure was modelled from the PDB structure 6EK0 with subsequent structural modification and simplifications using the software Maestro Schrodinger Release 2018-4 - academic version. The sequence of RPL10L (Uniprot: Q96L21) was manually modelled with the graphical user interface to provide the 3D sequence of RPL10 (Uniprot: P27635) within the 3D model. Likewise, mutations R98S and Q123R were obtained by mutating residues from the graphical user interface followed by a local minimization.

### Clustering analysis

The cluster heatmap was generated by analyzing all the genes recurrently mutated in T-ALL and genes encoding for i) proteins involved in ribosome biogenesis and translation and ii) RPs, as long as they are mutated in at least one patient. We considered them as mutated genes regardless of their germinal or somatic status. Jaccard’s distance was used to calculate the distance matrix and an Average Linkage Method was chosen through the maximization of the Cophenetic Correlation Coefficient. Analyses were performed with software R4.1.2 with the library pheatmap 1.0.12.

### Cells isolation and culturing conditions

BM or PB mononuclear cells (MNC) were collected by Ficoll-Paque centrifugation, washed twice in culture medium and then cryopreserved in FBS with 10% DMSO. All specimens consisted of 40%–100% of blasts. Frozen cells were thawed and diluted with RPMI (Corning) supplemented with 50% FBS and washed. Washed cells were resuspended in preheated RPMI and incubated at 37°C in a 5% CO_2_ humidified incubator for two and a half hours before the treatment for the SUnSET assay.

### SUnSET assay and western blot

Protein synthesis in patient’s cells was measured by SUnSET assay as previously described (Schmidt et al., 2009). Total proteins were extracted using RIPA buffer and resolved by SDS-PAGE with the stain-free technologies (TGX Stain-Free Fastcast Acrylamide kit, Bio-rad). The used primary antibodies are listed in supplementary methods.

### Phosphoflow cytometry assay

Thawed MNCs were assessed for count and viability by Trypan blue dye exclusion before phosphoflow testing as described previously (Bonaccorso et al., 2020). Please refer to the Supplementary methods for antibodies and further details. Positivity threshold for phosphoprotein expression was established by the comparative use of isotype IgG instead of the phosphoprotein specific antibody. Basal level of each phosphoprotein was calculated as % of phosphoprotein positive cells and as fold change (Supplementary Table S4).

## Results

### Whole exome sequencing outcome

**FIGURE 1:**
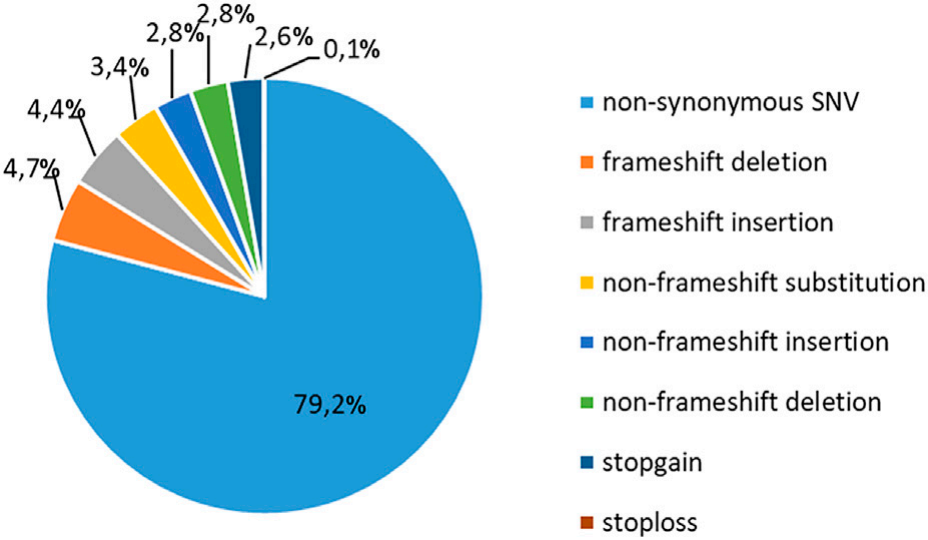
Classification of mutations in T-ALL patients. Frequency of recurrence of different subgroups of genetic alterations in 37 pediatric T-ALL samples. SNV: single-nucleotide variation.

To study the occurrence of RP mutations in T-ALL, we performed WES on the blood samples collected from 37 pediatric patients at the diagnosis of T-ALL (Supplementary Tables S1, S2). The sequencing analyses were performed at an average coverage of 79,27X (range 55,90- 104,91) and 92.59% of bases were covered by at least 20 reads (Supplementary Table S5). We identified a total of 25936 alterations, with a mean of 700,9 alterations (range: 470–1067) per case (Supplementary Table S6). The most frequent (79,2%) genetic alteration was represented by nonsynonymous single-nucleotide variants (SNVs), which likely include a number of genetic variants that cannot be classified as genetic lesions. The second most frequent alteration was represented by frameshift insertions and deletions (INDELs, 9,1%), nonframeshift INDELs (5,6%), nonframeshift substitutions (3,6%) and stopgain/stoploss mutations (2,7%, Figure 1).

### Overview of the identified genetic alterations

**FIGURE 2:**
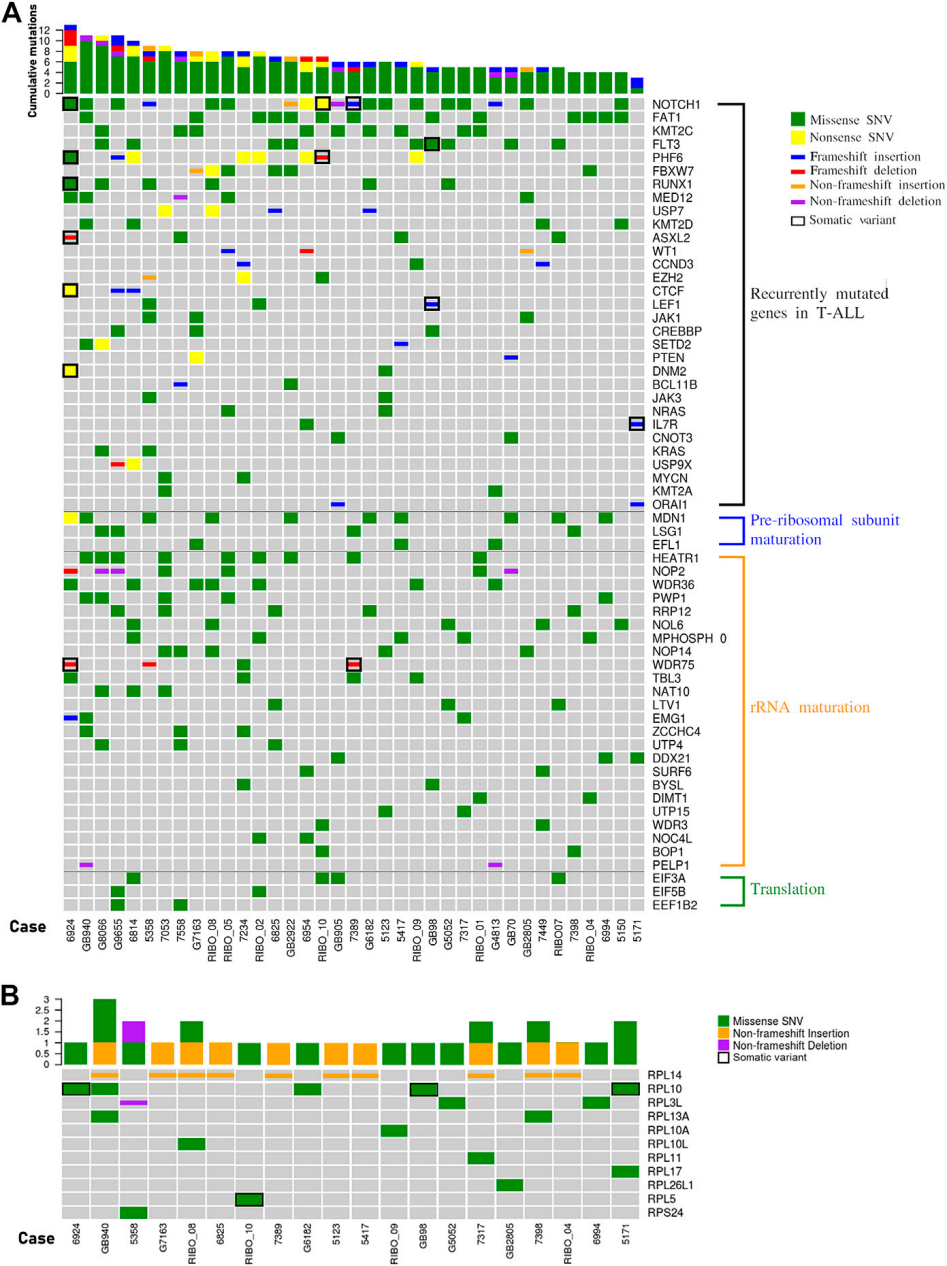
The landscape of mutations in pediatric T-ALL samples. **(A)** overview of variations in T-ALL driver genes (recurrently mutated genes in T-ALL) and other genes involved in ribosome biogenesis (pre-ribosomal subunit maturation and rRNA maturation) or in translational control. In the graphs only genes harbouring variations in at least two patients are represented. The total number of mutations per patient is represented by histograms at the top of the heatmap. **(B)** Overview of variations in ribosomal proteins. In the graph only those samples with at least one variation in genes encoding for ribosomal protein are reported. The total number of RP mutations per patient is represented by histograms at the top of the heatmap. SNV: single nucleotide variant.

We analyzed the WES data searching for mutations recurring in at least 2 patients in the cohort, and identified alterations in 31 of the most commonly mutated genes in T-ALL (Figure 2A). For 11 genes the mutation frequency was >10% and for 7 genes was >15%. The latter includes NOTCH1 (51,4%), FBXW7 (16,2%), PHF6 (21,6%), RUNX1 (16,2%), FLT3 (24,3%), KMT2C (24,3%), FAT1 (35,1%) (Figure 2A). In addition, defects in genes encoding for proteins and factors involved in ribosome biogenesis or in translational control have previously been identified as lesions driving the oncogenic process in T-ALL (de Keersmaecker et al., 2013; Liu et al., 2017). Therefore, we specifically focused on these sub-groups of genes, and divided them in 4 different functional groups: 1. RPs, 2. factors involved in pre-ribosomal subunits maturation, 3. factors involved in rRNA maturation, and 4. translation factors (Figure 2). Collectively, we found alterations in 41 genes belonging to these 4 categories. In particular, most patients showed alterations in factors involved in rRNA maturation and for 3 of these the frequency was higher than 15%: HEATR1 (24,3%), NOP2 and WDR36 (18,9%). Although most of the mutated factors belonged to that category, it is worth mentioning that the gene with the highest alteration frequency was MDN1 (27%) which belongs to the pre-ribosomal subunits maturation category. The alterations found in RPs–encoding genes are described separately in the next paragraph.

### Mutations in genes encoding for ribosomal proteins

Our NGS analysis identified a total of 32 sequence variations in 13 RPs genes in 23 patients (62,2%), and we confirmed 26 of them in 20 patients by Sanger sequencing (Figure 2B and Supplementary Table S7). While alterations in RPL10 (13,5%), RPL13A (5,4%), RPL14 (27,03%) and RPL3L (8,1%) were observed in two or more samples at diagnosis, alterations in other RPs were observed in one patient (Figure 2B). We then analyzed the matched remission samples of the patients in whose RPs alterations were detected, to evaluate their status (somatic vs. germline). This allowed us to call as somatic the p.G201D (c.G602A) in RPL5 gene (1 patient) and p.Q123R (c.A368G) and pR98S (c.C292A) in RPL10 genes (3 patients) (Figure 2B; Supplementary Figure S2). Furthermore, we observed a high variability in the number of repeats of a CTG triplet in exon 6 of RPL14 gene. This condition was germline for most patients, and somatic for one case, suggesting that it may be a microsatellite. Mutations in position 123 of RPL10 protein had already been identified (de Keersmaecker et al., 2013); however, the substitution of Glutamine with an Arginine had never been described before. Therefore, we decided to study in more detail the effect of this alteration.

**FIGURE 3:**
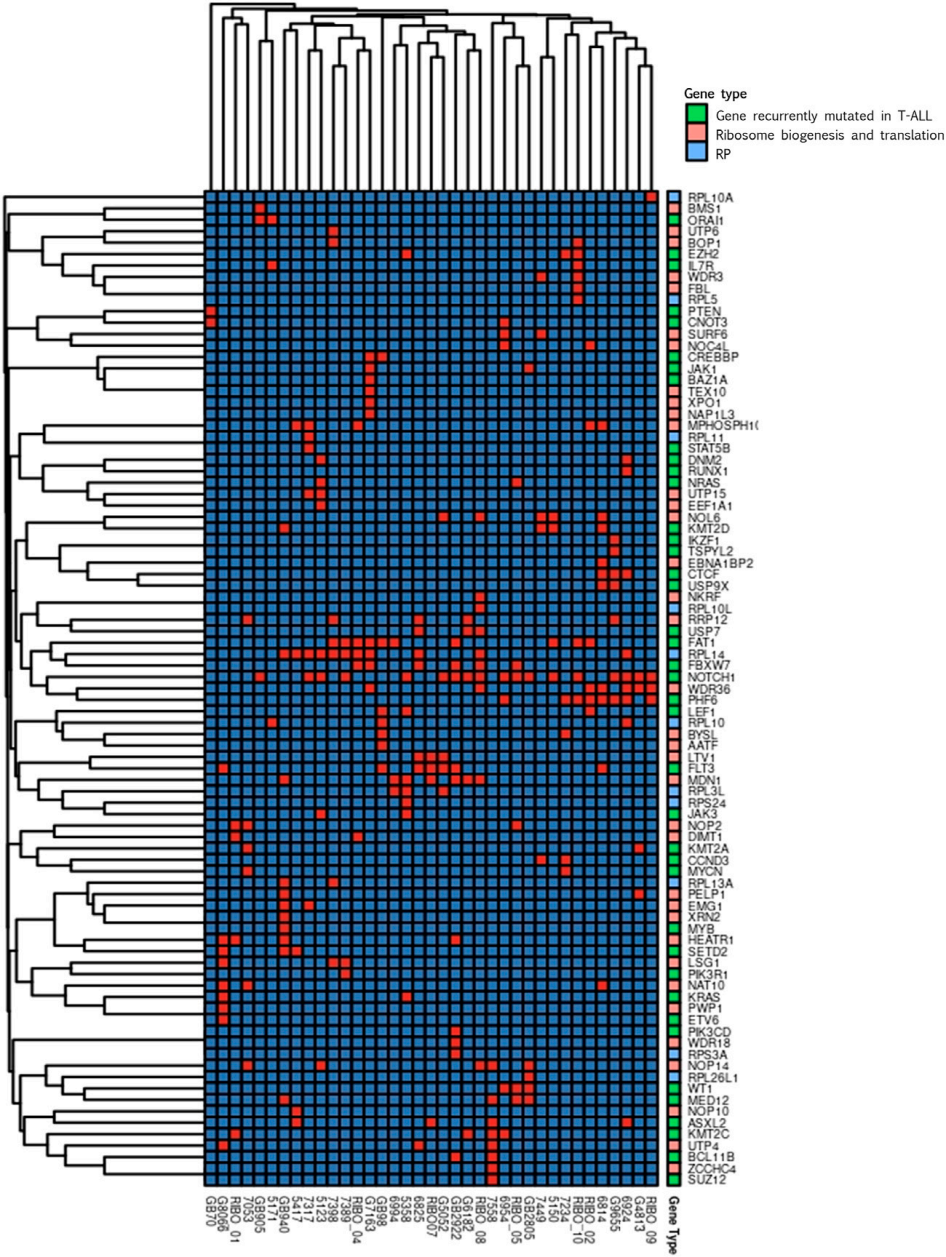
Clustering analysis. Cluster heatmap of variations detected in genes encoding for proteins involved in ribosome biogenesis and translation (pink), for ribosomal proteins (RP, light blue) and in genes recurrently mutated in T-ALL (green). In the graphs only genes harbouring variations in at least one patient are represented. Columns represent single patients; rows represent single genes. The order of both the patients and the genes is based on hierarchical clustering with the Jaccard distance. Presence of gene’s variation is indicated in red and the absence in blue.

We performed a clustering analysis of the genetic alterations to address the tendency of specific genes to co-mutate in the same patient. This could be a preliminary indication of compensative or mutually exclusive events in the oncogenic process. The results of the clustering analysis (Figure 3) indicated that most of the gene clusters tended to be supported by a few or even one single patient, conferring a low significance to those clusters.

Worth noting are the three groups of genes found co-mutated in at least three patients. One of these clusters included FAT1 and RPL14 that co-mutated in four patients, RPL14 co-mutate also with FBXW7 in four patients, two of which share also the FAT1 mutation. In the same cluster, the known T-ALL driver NOTCH1 was present. In addition, NOTCH1 was part of a second cluster with WDR36 and PHF6, that were co-mutated in four patients. These two genes are both involved in ribosome biogenesis, and PHF6 is recurrently found mutated in T-ALL. Interestingly, RPL14 and WDR36 were found co-mutated in 3 different patients, generating a representative macro-cluster. The third cluster encompassed LTV1 and FLT3 that were co-mutated in three patients.

### Modelling of RPL10 Q123R mutation

**FIGURE 4:**
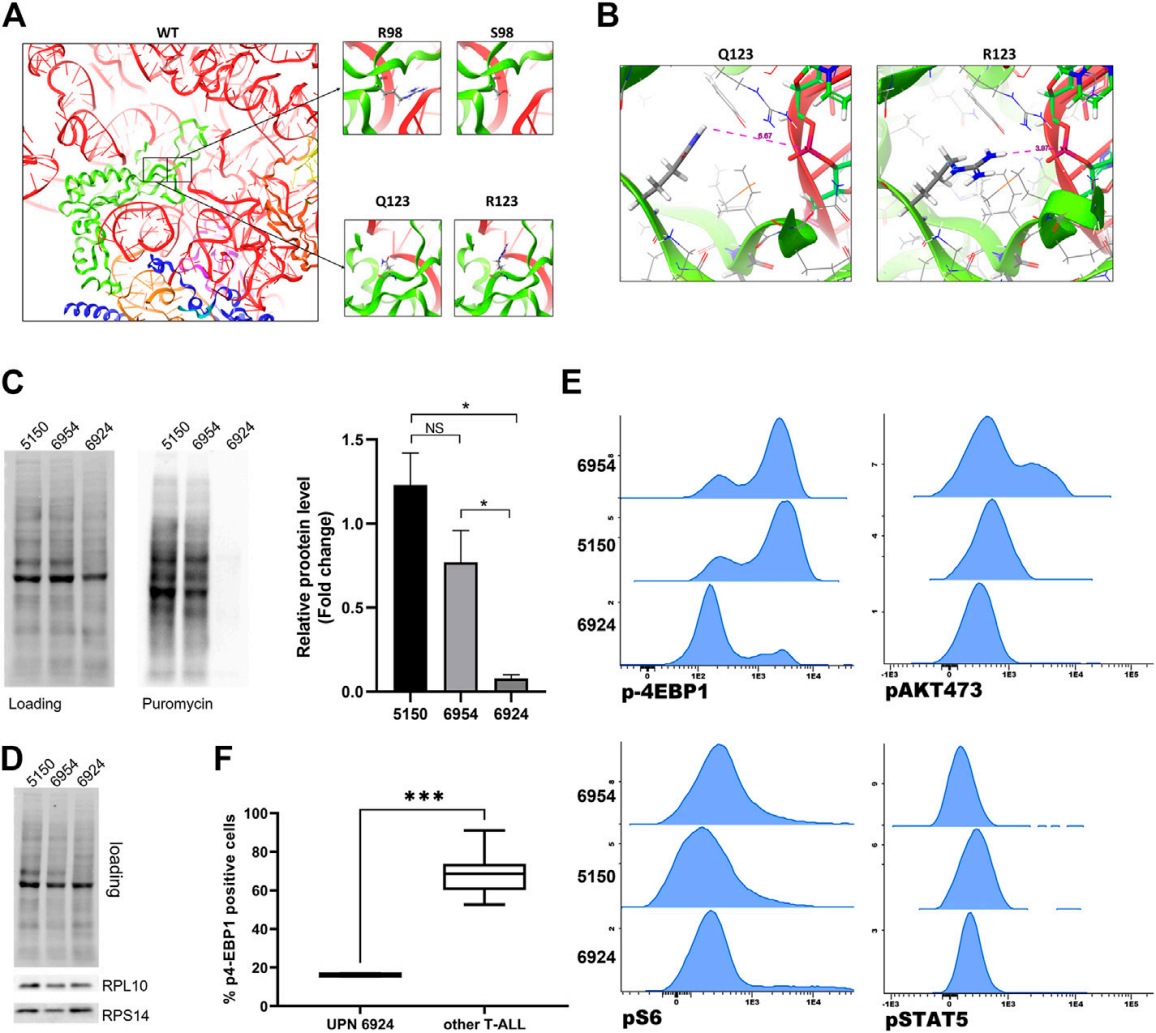
Modelling and functional study of RPL10 Q123R. **(A)** Representation of human RPL10 structure (in green) inside the ribosome, in proximity with RNA molecules (in red and light orange) and other chains such as RPL5 (in blue), RPL13 (in purple) and RPL36A (in dark orange). The black boxes represent the position of the residues R98 and Q123. The figure show that these residues are close to the ribosomal catalytic center. On the right, the amino acids substitutions R98S and Q123R are represented. WT: wild type; R: arginine; Q: glutamine; S: serine. **(B)** Predicted structure of the RPL10 Q123R mutation in relation to bystander ribosome components. The distances are measured from the side chain of the R to the 28S rRNA U1866. The model is based on PDB structure 6EK0. **(C)** Western blotting of SUnSET assay. On the left, representative experiment. SDS-PAGE separation was achieved on a 10% acrylamide gel. On the right, average of densitometric analyses of two independent experiments reported as fold change over an average of control samples (5150 and 6954, from patients with WT RPL10); 6924 is the sample from the patient bearing the RPL10 Q123R mutation. N = 2 experiments, *p < 0.05 by unpaired t test. NS: not significant. **(D)** Representative western blot of RPL10, RPS14 in the same patients. **(E)** Basal expression of p4EBP1, pAKT, pS6 and pSTAT5 was measured by phosphoflow in three T-ALL samples; histograms represent MFI of each phosphoprotein and indicate a significant downregulation of p4EBP1 in patient 6924, Q123R RPL10 mutated, compared to the control patients (5150 and 6954). No statistically significant differences in pS6, pAKT 473 and pSTAT5 expression were found. **(F)** Box plot distribution of p4-EBP1 in cells from Q123R RPL10 mutant and 7 non-mutant patients (***p = 0.0006 by unpaired t-test).

Q123 (as R98) forms the basis of an essential flexible and dynamic loop close to the catalytic core of the ribosome, which is involved in ribosome rotation during translation (Pollutri and Penzo, 2020). To study the effects of these amino acid substitutions in RPL10 structure, we generated a three-dimensional model of the 80S ribosomal structure, incorporating RPL10 R98S or Q123R (Figure 4A) mutations. The model showed that the replacement of a glutamine with an arginine, thus passing from a polar and neutral aminoacid to a positive charged one, may result in a relevant structural modification of RPL10 within the ribosome. This change could ultimately alter the interactions withheld by RPL10 with bystander proteins or rRNAs within the ribosome. Indeed, we envision a reduction in the distance between the side chain of the arginine amino acid and the ribosomal U1866 (Figure 4B).

### *Ex-vivo* functional study of RPL10 Q123R mutation

Given the known impact of R98S in T-ALL cells in terms of cellular proliferation, ribosome biogenesis, cell metabolism and resistance to pro-apoptotic stimuli (de Keersmaecker et al., 2013; Girardi et al., 2018; Kampen et al., 2019a; Kampen et al., 2019b), and based on our modelling of the Q123R RPL10 structure (Figure 4), we posited that the Q123R mutation could also have a role in T-ALL. Even though we did not have available an internally controlled model for the study of the effects of the mutation in leukemia cells (such as a knock-in mutant in a T-ALL cell line), we decided to observe some specific phenotypic features of patient’s cells bearing the RPL10 Q123R mutation, compared to other patients of the same cohort. One of the possible general readouts of ribosome function is the rate of protein synthesis. Therefore, to test our hypothesis, we investigated the impact of Q123R mutation on the protein synthetic activity in leukemic cells. As shown in Figure 4C, protein synthesis in these cells was strikingly reduced, compared to the protein synthesis rates of leukemic cells from other patients of our cohort, not bearing the RPL10 Q123R mutation. These patients were selected as controls based on their similar (albeit not identical) mutational landscape, specifically regarding the major known driver genes, and were missing any RP alterations (Figure 2).

Nonetheless, the abundance of ribosomes in these cells was comparable, as we found equal amounts of different large and small subunit RPs (Figure 4D), indicating that the difference in protein synthesis observed in RPL10 Q123R mutated cells could not be explained with a lower availability of ribosomes.

We then examined the phosphorylation status of JAK/STAT and PI3K/AKT pathway proteins by phosphoflow, including eIF4E-binding protein 1 (4EBP1, a known regulator of translation). As depicted in Figure 4E, and in keeping with the results shown in Figure 4C, p-4EBP1 was significantly downregulated in patient 6924 bearing the RPL10 Q123R mutation as compared to the two control patients (5150 and 6954). Similarly, the phosphorylation level of 4EBP1 in patient 6924 was significantly lower also when compared to the other five T-ALL patients of our cohort (5123, 7053, 7389, 7449 and 7558) (Figure 4F). However, no statistically significant differences were found in pS6, pAKT 473 and pSTAT5 phosphorylation levels (Figure 4E; Supplementary Figure S3).

## Discussion

The outcome of T-ALL in children has improved over the past decades. However, 15% of patients never reach long term remission, due to failure of currently available therapies (Lato et al., 2021). Therefore, current research efforts are focusing on identifying new targets for the development of more effective and less toxic personalized therapies, a strategy which requires a detailed understanding of the genetics and biology of this disease.

In recent years, somatic lesions affecting RPs, ribosome biogenesis, or factors involved in translation processes, have been described in a wide variety of human hematologic and solid tumors (Stumpf and Ruggero, 2011; Sulima and de Keersmaecker, 2017). The cancer promoting action of such lesions is supported by the observation that patients with congenital mutations in RPs or ribosome biogenesis factors develop ribosomopathies, diseases often characterized by hypo-proliferative phenotypes (like hemopoietic insufficiency) early in life, and, later by an increased risk of developing hematopoietic malignancies and solid tumors [rev. in (Kampen et al., 2020)].

Recent sequencing-based studies on infant T-ALL highlighted the presence of somatic mutations in RPL5, RPL10, RPL11 and RPL22, with variable frequency (Rao et al., 2012; de Keersmaecker et al., 2013; Tzoneva et al., 2013).

Given that ribosome biogenesis and translation are very complex processes, with our analysis we aimed to understand if mutations in RP-encoding genes may be correlated with other alterations in genes involved in the same processes, even though the size of our cohort was relatively small, thus not allowing for a high statistic power. Nonetheless, the frequency of the mutations detected in known T-ALL driver genes was similar to those previously reported in other studies. For example, the frequency of NOTCH1 mutation in our cohort was 51%, consistent with what reported in (de Keersmaecker et al., 2013), (Girardi et al., 2017) (43%) (Liu et al., 2017) (>70%) and (Zhang et al., 2012; McLeod et al., 2021) (61%). The latter cohort, available in the Pecan database, is particularly interesting since it is of relevant size (426). As reported in Supplementary Table S8, we found that most of the genes of interest showed in Figure 2A were reported to be hit by somatic mutations in Pecan T-ALL cohort, and for most (but not all) of them the frequencies of occurrence in the two cohorts did not differ significantly. This may indicate that, for some genetic alterations, the frequencies of occurrence in our cohort may be overestimated due to the small sample size. We found somatic mutations in RPL10 gene with a frequency of 8%, in line with what was previously found in a larger cohort by de Keersmaecker et al. (2013); Liu et al., 2017. It is worth noting that, while mutations in other RPs have been described in multiple cancer types, mutations in RPL10 have been mainly found in pediatric T-ALL (Goudarzi and Lindström, 2016; Brady et al., 2022). Interestingly, the RPL10 residues hit by the mutations are also recurring, namely R98 and Q123. R98 is a known mutational hotspot in pediatric T-ALL, and the R98S substitution accounted for two thirds of the somatic mutations detected in RPL10 in our cohort. The Q > P substitution in position 123 has been reported with a much lower frequency (de Keersmaecker et al., 2013; Liu et al., 2017), and here we describe for the first time the Q123R somatic substitution. Q123 lies adjacent to R98 within the protein 3D structure; both residues are at the base of a flexible loop that is in close proximity to the peptidyl-transferase center in ribosomes’ catalytic core (Ben-Shem et al., 2011). A mutation in this site may alter the flexibility of the loop, ultimately affecting ribosomal function. Previous studies performed in yeast and humans have demonstrated that the RPL10-R98S mutation alters translational efficiency and fidelity (de Keersmaecker et al., 2013; Sulima et al., 2014). Due to its position in the functional core of the ribosome, it is not surprising that mutations in R98 may impinge on ribosomal structure and activity. It is proven that R98S impacts not only cellular proliferation and ribosome biogenesis (de Keersmaecker et al., 2013), but also leukemic cell metabolism (Kampen et al., 2019a), resistance to pro-apoptotic stimuli (Kampen et al., 2019b), and cross-talk with known leukemia driver pathways, like JAK-STAT (Girardi et al., 2018). While the contribution of R98S RPL10 mutation to pediatric T-ALL has been extensively studied, nothing is known about the contribution to leukemia of other RPL10 mutations, like the ones involving Q123. Mutations involving R98 and Q123 could lead to similar functional outcomes, and our modeling approach, indeed, showed that the replacement of glutamine with arginine, two amino acids with different chemical characteristics (polar vs. basic), has the potential to structurally alter the spatial organization of RPL10 flexible loop within the ribosome. Based on our model, protein-rRNA interactions in the catalytic core of the ribosome may be modified, thus possibly affecting specific catalytic features. To investigate such hypotheses, we assessed the protein synthetic capacity of leukemic cells obtained from the patient with the Q123R RPL10 mutation. Strikingly, we found extremely poor protein synthesis in the mutant sample compared to controls. This result could be a composite effect of a reduced translational capacity of RPL10 Q123R ribosomes and of other factors (altered transcription, altered upstream signaling, etc.). Indeed, our phosphoflow analysis of the activation of known oncogenic phosphorylation cascades, indicated a down-regulation of the phosphorylation of 4EBP1 in the patient carrying the Q123R RPL10 mutation. It is known that 4EBP1, as a substrate of the mechanistic target of rapamycin (mTOR) signaling pathway, is the main regulator of eIF4E availability. In the hypophosphorylated state, 4EBP1 competes with eIF4G for binding to eIF4E, thus resulting in the inhibition of the formation of the translation pre-initiation complex (PIC) (Musa et al., 2016). Therefore, a combination of the inhibition of the PIC and possibly a lower processivity of the Q123R mutant ribosome could lead to a substantial reduction of translation, as observed.

These results suggest that RPL10 Q123R could have a role in T-ALL, even though the molecular and phenotypic effects triggered by this mutation need to be dissected in more appropriate models, such as isogeneic mutant/WT cell lines. Nonetheless, they may be in keeping with the consolidated concept that cancer cells find and select for alterations, often converging on translation, that help them to resist stressing stimuli (e.g. oxidative stress, metabolic stress, nucleolar stress, etc.) (de Keersmaecker et al., 2013; Schwarzer et al., 2015; Girardi et al., 2018; Kampen et al., 2019b; Kampen et al., 2019a; Penzo et al., 2019; Kovalski et al., 2022). Reduced protein synthesis found in the mutated sample could also impact the response to therapy of T-ALL. Indeed, some conventional therapeutic schemes for ALL based on glucocorticoids and other cytotoxic drugs, are more effective on highly proliferating cells (Ebinger et al., 2016).

In addition to the mutations found in RPL10, our analyses highlighted the presence of genetic alterations in RPL3L, RPL5, RPL10A, RPL10L, RPL11, RPL13A, RPL14, RPL17, RPL26L1 and RPS24. Most of these sequence variations turned out to be germline alterations, with no known pathogenetic effect, except for p.S202N (c.G605A) in RPL10, which had previously been associated with intellectual disability (Klauck et al., 2006; Brooks et al., 2014). It is worth noting that the patient of our cohort bearing this mutation had a normal intellectual development. In addition, the somatic substitution p.P172L (c.C515T) in RPL3L has previously been associated with melanoma (Wagle et al., 2014). RPL14 deserves a special mention, since it is characterized by the presence of a variable number of CTG (encoding for leucine) triplets, as also reported on the NCI database for single nucleotide polymorphisms (dbSNP). All these variants are, however, of benign significance. Even though there are other examples of such triplet expansion/contraction in other genes, to the best of our knowledge this feature is unique to RPL14 among RP encoding genes.

Mutations in RPL5 have previously been reported in childhood T-ALL (de Keersmaecker et al., 2013; Liu et al., 2017; Brady et al., 2022), and we report a mutation frequency in line with other T-ALL cohorts (2%) (de Keersmaecker et al., 2013). While RPL10 somatic mutations concentrate in two mutational hotspots, and mainly consist of amino acid substitutions, in the case of RPL5 these are of different nature (frameshift, substitution, nonsense) and are spread all over the protein (de Keersmaecker et al., 2013; Liu et al., 2017; Brady et al., 2022). This specific characteristic makes it more difficult to mechanistically study the effects of RPL5 mutations in T-ALL. The mutation of RPL5 that we report here, the G201D substitution, has never been described before, not only in T-ALL, but in general in human cancer. However, a G201V RPL5 mutation has been described in malignant melanoma (Tate et al., 2019).

We tackled the connection between the occurrence of mutations in genes involved in ribosome biogenesis and translation, and those found more frequently in T-ALL. Because alterations affecting translational machinery may not be well tolerated by cells, our interest was specifically in assessing whether there may be recurrently co-occurring genetic alterations, which could have compensatory effects. Indeed, we found that there are some genetic aberrations that present together in several patients of our cohort. These involve genes which are known to be leukemia drivers (such as FAT1, NOTCH1, PHF6, FLT3), and can activate signaling pathways that push the cells to multiply faster, as well as genes involved in ribosome biogenesis or translational control (such as WDR36 and LTV1). We thus speculated that the latter alterations may contribute to tune down the rate of protein synthesis, bringing it down to levels which that are bearable for the cells.

In conclusion, our study indicates that genetic alterations hitting genes involved in ribosome biogenesis and translational control should be carefully considered in the context of T-ALL. Although the functional outcome of most of these mutations is overlooked at the moment, mainly due to lack of mechanistic knowledge, future studies will be needed to explore the effect of the co-existence of such mutations. In the rising era of precision medicine, this knowledge will be essential to provide the patients with the best and most tailored therapeutic options.

## Data Availability

The data presented in the study are deposited in the BioProject repository, accession number PRJNA876791. The link and detailed accession numbers are listed in Supplementary Material.

## Supplementary material

Supplementary Figure S1 Gating strategy used to identify T-ALL cells for phosphoflow analyses. Upper: representative density plot on CD45low/CD7+ blasts and CD45high/CD7+ residual normal T lymphocytes. Lower: representative density plot expressing % of p4EBP1 in a patient carrying RPL10 Q123R mutation (6924) and a patient RPL10 wt (6954).

Supplementary Figure S2 Sanger results for RPL10 and RPL5 mutations. Representative chromatograms of the presence of the RPL10 or RPL5 mutations in diagnosis or remission genomic DNA.

Supplementary Figure S3 Basal expression of phosphoprotein profiling in T-ALL samples. Distribution of % positive cells (with median) were measured for each phosphoprotein in 8 T-ALL samples and are represented in a scatter plot graph. Red dots: 6924 T-ALL patient with the Q123R RPL10 mutation (two independent experiments). Black dots: 5150, 6954, 5123, 7053, 7389, 7449 and 7558 T-ALL patients with wild type RPL10.
