# Supplementary figures and images for: Mutational analysis of ribosomal proteins in a cohort of pediatric patients with T-cell acute lymphoblastic leukemia reveals Q123R, a novel mutation in RPL10
Source: Front Genet. 2022 Nov 22;13:1058468. doi: 10.3389/fgene.2022.1058468 (PMC9723238; doi:10.3389/fgene.2022.1058468)

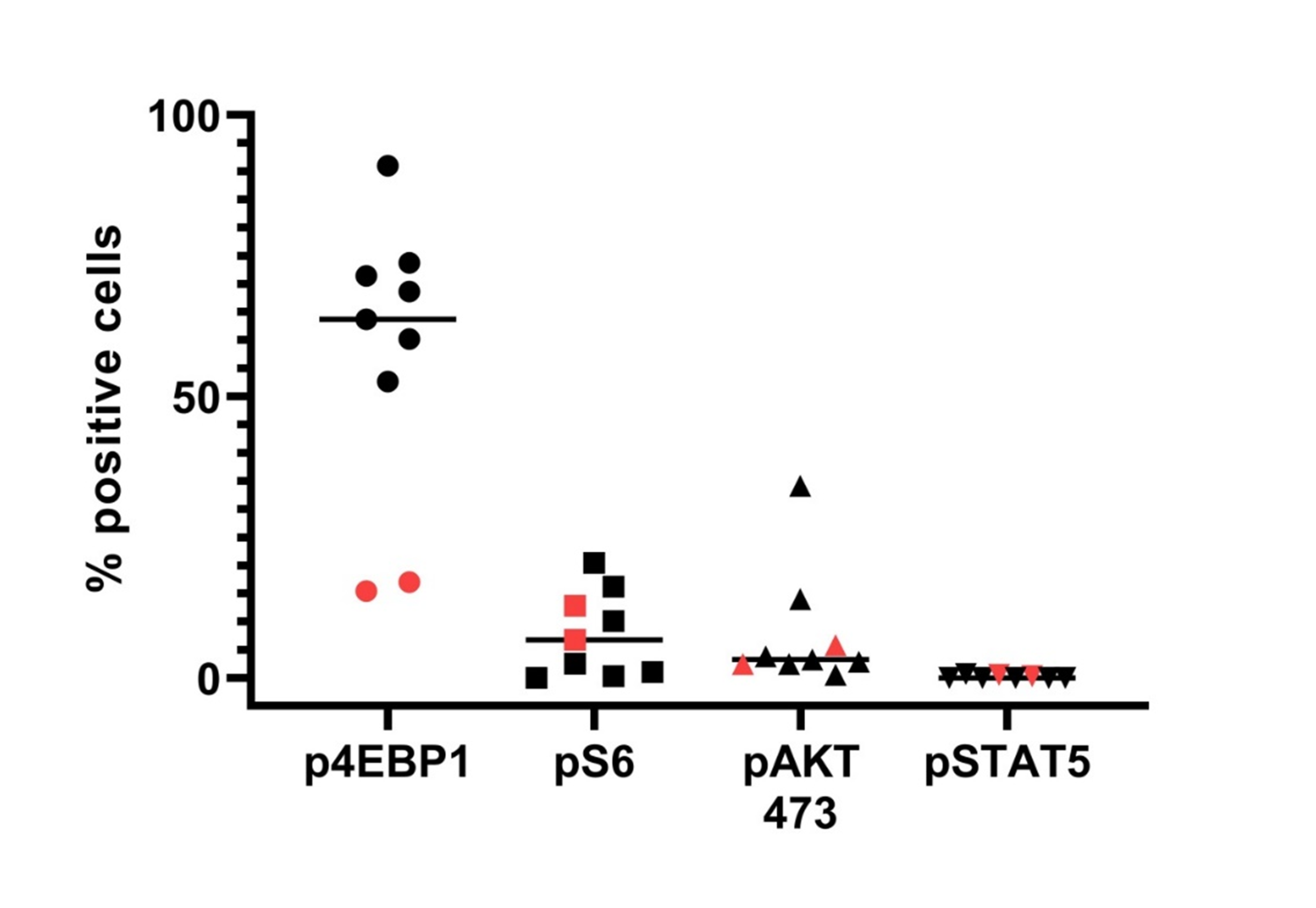

Supplement: Supplementary file 1 [file Image3.TIF]

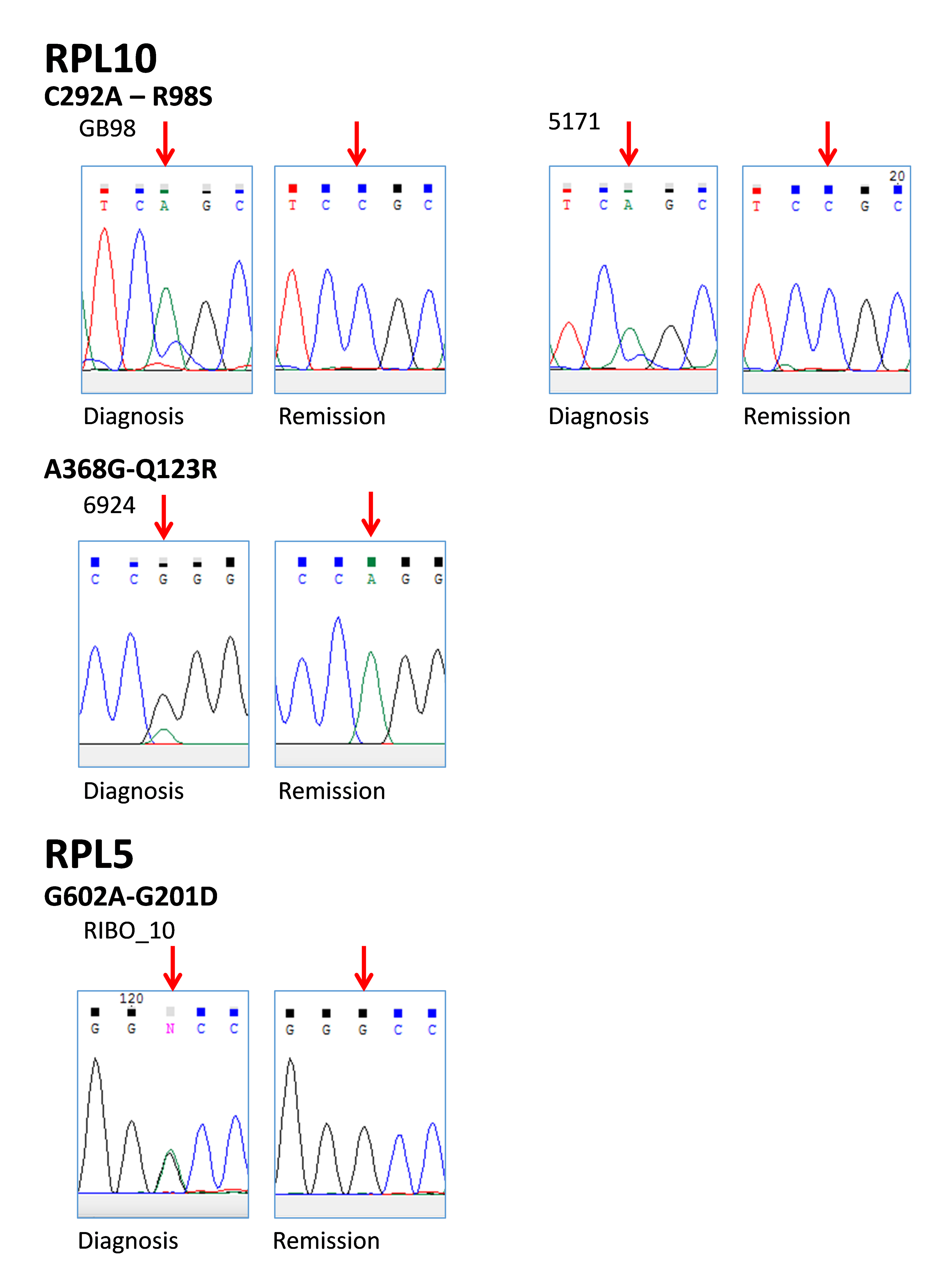

Supplement: Supplementary file 2 [file Image2.TIF]

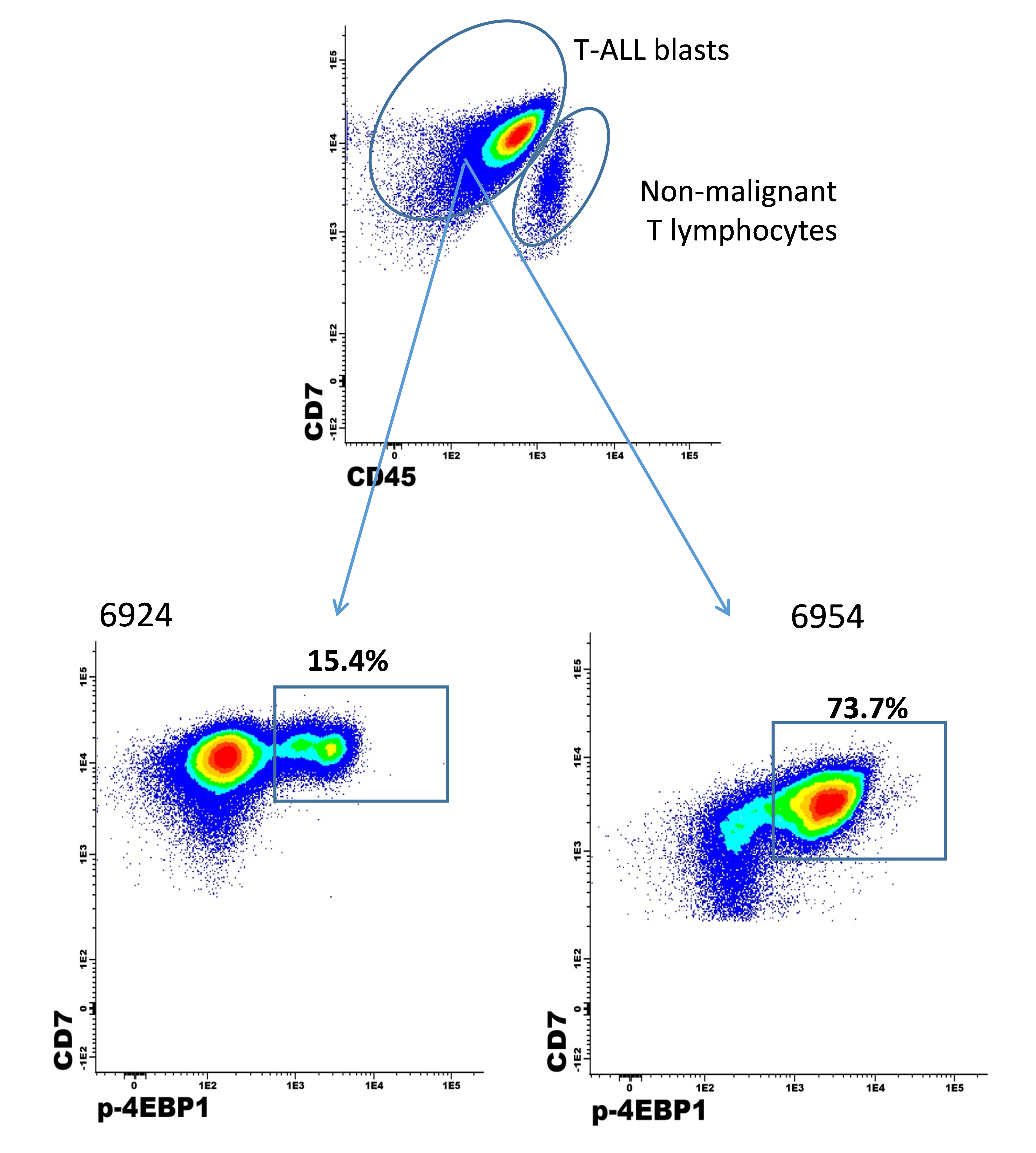

Supplement: Supplementary file 3 [file Image1.TIF]
