# Supplementary material for: Mutational analysis of ribosomal proteins in a cohort of pediatric patients with T-cell acute lymphoblastic leukemia reveals Q123R, a novel mutation in RPL10
Source: Front Genet. 2022 Nov 22;13:1058468. doi: 10.3389/fgene.2022.1058468 (PMC9723238; doi:10.3389/fgene.2022.1058468)
Supplement: Supplementary file 5 [file DataSheet2.docx]

**Supplementary Materials**

**Antibodies**

The following antibodies were used for western blot analysis: puromycin (Merck MABE343, 1:5000), RPL10 (Abcam ab138978, 1:500), RPS14 (Santa Cruz Biotechnology sc-68873, 1:500).

**Phosphoflow cytometry assay**

Briefly, thawed cells were starved in X-Vivo medium, then fixed with 1.5% paraformaldehyde and permeabilized with 90% ice-cold methanol prior to staining with anti-phospho-protein-directed monoclonal antibodies: p-4EBP1 Alexa 488, p-AKT473 Alexa 647, p-S6 Alexa 647 (all from Cell Signaling), p-STAT5 Alexa 488 (BD) or isotypes matched IgG and surface antigen-directed anti CD7 ECD (Beckman Coulter) and anti CD45 PerCP (BD). Cells were acquired on SLRFortessa™ X20 flow cytometer (BD). Data were collected and analyzed using the DIVA™ (BD) and Infinicyt v2.0 (Cytognos) softwares. Leukemic cells and normal residual T-cells were identified by using CD7-ECD and CD45-PerCP antibodies as shown in Suppl. Figure 1.
